# Sourdough Fermentation Favorably Influences Selenium Biotransformation and the Biological Effects of Flatbread

**DOI:** 10.3390/nu10121898

**Published:** 2018-12-03

**Authors:** Mattia Di Nunzio, Alessandra Bordoni, Federica Aureli, Francesco Cubadda, Andrea Gianotti

**Affiliations:** 1 Department of Agri-Food Sciences and Technologies (DISTAL), University of Bologna, Piazza Goidanich 60, 47521 Cesena, Italy; mattia.dinunzio@unibo.it (M.D.N.); andrea.gianotti@unibo.it (A.G.); 2 Department of Food Safety, Nutrition, and Veterinary Public Health, Istituto Superiore di Sanità-Italian National Institute of Health, Viale Regina Elena 299, 00161 Rome, Italy; federica.aureli@iss.it (F.A.); francesco.cubadda@iss.it (F.C.)

**Keywords:** sourdough fermentation, selenium, in vitro digestion, HepG2 cells

## Abstract

Although selenium is of great importance for the human body, in several world regions the intake of this essential trace element does not meet the dietary reference values. To achieve optimal intake, fortification of bread by using selenium-enriched flour has been put forward. Less is known on the potential effect of sourdough fermentation, which might be worth exploring as the biological effects of selenium strongly depend on its chemical form and sourdough fermentation is known to cause transformations of nutrients and phytochemicals, including the conversion of inorganic selenium into organic selenocompounds. Here we investigated the bio transformation of selenium by sourdough fermentation in a typical Italian flatbread (piadina) made with standard (control) or selenium-enriched flour. The different piadina were submitted to in vitro digestion, and the biological activity of the resulting hydrolysates was tested by means of cultured human liver cells exposed to an exogenous oxidative stress. The use of selenium-enriched flour and sourdough fermentation increased the total content of bioaccessible selenium in organic form, compared to conventional fermentation, and led to protective effects counteracting oxidative damage in cultured cells. The present study suggests that selenium-rich, sourdough-fermented bakery products show promise for improving human selenium nutrition whenever necessary.

## 1. Introduction

Selenium (Se) is an essential trace element critical to the normal physiology of a wide range of species, including humans [1]. As a part of l-selenocysteine (SeCys, the 21st amino acid), Se is needed for the synthesis of selenoproteins, a class of proteins with important functions including antioxidant defense, T-cell immunity, thyroid hormone metabolism, and skeletal and cardiac muscle metabolism [2]. The liver is the key site of human Se metabolism and synthesizes most of plasma selenoprotein P, thereby regulating whole-body Se transport and homeostasis. Se deficiency affects the expression and function of selenoproteins and has been associated to increased risk of infertility and prostate cancer in men, nephropathy, neurological diseases, dilated cardiomyopathy (Keshan disease), and endemic osteoarthropathy (Kashin–Beck disease) [3,4]. There is evidence that adequate and supranutritional Se intake, maintaining full expression of selenoproteins, may be beneficial with respect to the prevention of cancer. However, the relationship between selenium and cancer is complex and the effectiveness of dietary Se supplementation appears to depend on many factors, including baseline Se status, age, gender, and genetic background of an individual; type of cancer; and time point of intervention in addition to type of applied Se compounds and dose [5,6,7,8,9].

Low Se areas are present in a number of countries worldwide and; therefore, in several world regions Se dietary intake does not meet the dietary reference values set on the basis of the optimization of plasma glutathione peroxidase (GPx) 3 activity (ca. 55 µg/day in adults) [10], or the leveling off of plasma selenoprotein P concentration (70 μg/day in adults) [11]. As a result, efforts have been made to identify strategies to improve human Se nutrition in areas where Se is in short supply in the food chain, including Se biofortification and the production of Se-enriched foods. In developing such strategies, it has to be kept in mind that the range of intake separating deficiency and toxicity is narrow and, thus, care has to be taken in identifying the optimal range for health whilst avoiding Se overexposure. The tolerable upper intake level is set at 300 μg/day for adults (60 μg/day for children 1–3 years) [11], even though recent evidence indicates that long-term exposure to such a level may represent a risk for human health [12].

When the production of Se-enriched foods is targeted, the bioavailability of the nutrient must be carefully considered [13]. Se bioavailability depends not only on the food matrix, but also on the Se chemical form [14]. In fact, Se is more bioavailable in its organic forms (e.g., Se-methionine (SeMet) and SeCys) [15], whereas inorganic Se (selenite and selenate), despite being well absorbed, is less retained in the body [16]. Therefore, organic forms of Se should be the preferred choice for food enrichment and population-wide supplementation strategies [17].

In bakery food, conversion from inorganic to organic Se can occur during sourdough fermentation [18]. Sourdough is characterized by a complex microbial ecosystem, mainly represented by lactic acid bacteria and yeasts. It confers to characteristic features of food products such as palatability and high sensory quality [19]. The microbiota of cereal sourdough is based on stable associations of lactic acid bacteria (LAB) and yeasts selected by the process conditions and environmental variables (i.e., humidity, temperature, and oxygen availability). The LAB species that is typically isolated in wheat and rye sourdough belongs to the *Lactobacillus* genus, which represent the main component of the microbial consortium [20].

The aim of the present study was to verify whether sourdough fermentation, besides its well-known positive impact on organoleptic and functional properties of food products (e.g., synthesis of exopolysaccharides, increase of bioactive compound availability, decrease of antinutritional compounds, gluten detoxification by proteolysis, enrichment in vitamins, amino acids, and peptides, and glycemic index lowering due to acidification) [21,22,23,24], can effectively convert inorganic Se to organic species. A second objective was to test the biological activity of the latter in a real food matrix.

The typical flatbread of the Italian region, Emilia Romagna (piadina), was used as the model food. Flatbread contribute approximately 85% of the caloric value to the diet of Middle Eastern populations, and sourdough fermentation was already demonstrated to improve the shelf life of piadina [25]. Different piadina were prepared with control wheat flour or inorganic Se-enriched wheat flour and conventional (*Saccharomyces cerevisiae*-based) and sourdough fermentation were compared.

The different piadina were submitted to in vitro simulated human digestion [26,27], and the resulting hydrolysates were supplemented to cultured human liver cells (HepG2) exposed to exogenous oxidative stress.

## 2. Materials and Methods

### 2.1. Chemicals

Deionized water obtained by a Milli-Q Element System (Millipore, Molsheim, France) was used throughout. Ultrapure nitric acid 68% *v*/*v* was from Carlo Erba Reagenti (Milan, Italy) and ultrapure hydrogen peroxide 31% *v*/*v* was from Merck KGaA (Darmstadt, Germany). Calibrants and the internal standard (rhodium) for total Se measurements were from High Purity Standard (Charlestone, SC, USA). Analytical grade trimethylamine, acetic acid 99%, and ammonium acetate were from Sigma-Aldrich (San Diego, CA, USA), Baker Instra Analyzed, Avantor Material (Arnhem, The Netherlands), and Merck KgaA (Darmstadt, Germany), respectively. Dulbecco’s modified Eagle’s medium (DMEM) and Dulbecco’s phosphate-buffered saline (DPBS) were purchased from Lonza (Milan, Italy). All other chemicals and solvents were of the highest analytical grade and were obtained from Sigma-Aldrich (Milan, Italy).

All the lactic acid bacteria (LAB) and yeast strains used belonged to the culture collection of the Department of Agriculture and Food Science (DISTAL) of the University of Bologna. For the cultivation and counts of LAB, De Man, Rogosa, and Sharpe (MRS) medium (Oxoid, Basingstoke, Hampshire, UK), supplemented with maltose (5 g/L), yeast extract (5 g/L), and cycloheximide (0.1 g/L), was used. For the yeast strain, SDB (Sabouraud Dextrose Broth, Oxoid) medium, supplemented with chloramphenicol (0.1 g/L), was selected.

### 2.2. Piadina Preparation: Formulation, Fermentation, and Cooking

Italian refined durum wheat flour was used to prepare the piadina. Se enrichment was obtained by adding 5.66 mg Na_2_SeO_3_/kg dough before fermentation, which was obtained by either conventional or sourdough fermentation. For sourdough preparation a 10% (fresh weight basis—fwb) mixed-strain containing *Lactobacillus plantarum* 98A, *Lb. sanfranciscensis* BB12, *Lb. brevis* 3BHI, was added to the dough and fermented at 32 °C for 24 h to obtain a mature sourdough. The microbial load in mature sourdough was approximately 10^9^ colony forming units (CFU)/g for LAB and 10^7^ CFU/g for *S. cerevisiae* (pH around 3.5). For conventional fermentation, 3% (fwb) or 14% (fwb) of *S. cerevisiae* LBS was added to the final dough for 1.5 h of leavening at 32 °C. 

Four different types of piadina were obtained: (1) Control flour and conventional fermentation (CFCF); (2) Se-enriched flour and conventional fermentation (SFCF); (3) control flour and sourdough fermentation (CFSF); and (4) Se-enriched flour and sourdough fermentation (SFSF). All piadina were made in triplicate according to the same recipe (Table 1), then cooked on a hot plate (200 °C) for 45 s, each side.

### 2.3. In Vitro Digestion

Human digestion was simulated as described by Marcolini et al. [28] with slight modifications. In vitro digestion was performed in a 100 mL flask kept at 37 °C in a water bath on a magnetic stirrer equipped with a heating plate. Chemical composition of the digestive fluids, pH, and residence periods were adjusted to simulate the physiological conditions in the mouth, stomach, and small intestine phase, respectively. A buffer solution (120 mM NaCl, 5 mM KCl, and 6 mM CaCl_2_—pH 6.9) was added in proper volumes at every step. For 1 g of piadina dry matter, 2, 4, and 4 mL of buffer were added to resemble saliva, gastric juice, and duodenal juice, respectively.

To simulate mastication and oral digestion, 29.7 g of piadina (22 g of dry matter), obtained by pooling equal parts of the three independent food preparations, were ground with mortar and pestle and subjected to amylase digestion for 5 min, adding 44 mL of buffer solution containing 90 U/mL α-amylase. Then, 88 mL of buffer solution was added, and the pH was decreased to 2 by dropwise addition of 37% HCl. Gastric digestion was started by adding pepsin to a final concentration (f.c.) of 3 mg/mL. After a 60 min incubation period, 88 mL of buffer solution was added, and the pH was increased to 5 with 1.5 M NaHCO_3_ to stop peptic digestion. Duodenal digestion started with the addition of pancreatin (0.4 mg/mL f.c.) containing pancreatic lipase, amylase, ribonuclease, trypsin and other proteases, and bile (2.4 mg/mL f.c.). The pH was adjusted to 6.5 with 1.5 M NaHCO_3_, and digestion continued for an additional 180 min. Resulting gastro-intestinal (GI) hydrolysates were centrifuged at 4000 g for 5 min and at 21,000 g for 30 min, and the supernatant was filtered with 0.2 mm membranes (talis qualis digested sample—TQ). An aliquot of the TQ sample was sequentially ultrafiltered with the Amicon Ultra (Millipore, Milan, Italy) at a 3 KDa of molecular weight cut-off (L3 fraction, containing molecules with molar mass <3 KDa). Each type of piadina was digested in triplicate and equal amounts of hydrolysates were pooled together. Pooling was performed to reduce experimental variability associated to food preparation and digestion.

### 2.4. Determination of Se Content and Se Species

Total Se was measured in the TQ and L3 fractions. Oxidative digestion of the hydrolysates was performed using a Multiprep apparatus (FKV, Bergamo, Italy). Two mL of extracts were left overnight in 2 mL HNO_3_ (13 M), then added with 1 mL H_2_O_2_ and irradiated using a 1 h ramp up to 90 °C and then 6 h at 90 °C. Total Se measurements were carried out by inductively coupled plasma mass spectrometry (ICP-MS), using an Elan DRC II (PerkinElmer, Waltham, MA, USA) in the dynamic reaction cell mode, as detailed in Bhatia et al. [26]. 

The certified reference material NIST Durum Wheat Flour 8436 (National Institute of Standards and Technology, Gaithersburg, MD) was included in each analytical batch for quality control (trueness of 105%, precision of 5%).

Se species in TQ and L3 fractions were characterized by anion exchange high performance liquid chromatography (HPLC)-ICP-MS, as detailed elsewhere [26]. For the quantification of Se species, 1 mg/mL stock solutions, expressed as Se, were prepared by dissolving, in water, adequate amounts of selenious acid [Se(IV)], selenic acid [Se(VI)], seleno-l-methionine (l-SeMet), and methyl-seleno-l-cysteine (MeSeCys) (Sigma-Aldrich, San Diego, CA, USA). The exact concentrations of the stock solutions were ascertained by ICP-MS analysis. The purity of the standards was checked by HPLC-ICP-MS and no species interconversion was found. Se species were identified by retention time matching with the standard substances spiked to the samples. Quantitative calculations were based on peak areas. The performance characteristics of the method are given in Supplementary Table S1, and representative SI chromatograms are reported in Supplementary Figure S1 and Supplementary Figure S2.

### 2.5. Total Antioxidant Capacity of Gastro-Intestinal Hydrolysates

Total Antioxidant Capacity (TAC) was measured in the L3 fraction as described in Di Nunzio et al. [29], based on the ability of the antioxidant molecules in the sample to reduce the radical cation of 2,2′-azinobis-(3-ethylbenzothiazoline-6-sulfonic acid) (ABTS•+). TAC was determined in 10 μL of L3 fraction by evaluating the decoloration of ABTS•+, measured as the quenching of the absorbance at 734 nm. Values obtained were compared to the concentration–response curve of the standard trolox solution and expressed as mM of trolox equivalent (TE).

### 2.6. Cell Cultures, L3 Supplementation, and Oxidative Stress

HepG2 cells were maintained at 37 °C, 95% air, 5% CO_2_ in DMEM supplemented with 10% (*v*/*v*) fetal calf serum (FCS), 100 U/mL penicillin, and 100 µg/mL streptomycin [30]. Once a week cells were split 1:20 into a new flask, and the medium was refreshed. Cells were seeded in 6-well plates, and after 24 h at 75%–80% confluence, they were supplemented with the L3 fraction at 50 µL/mL medium concentration. Cells supplemented with 1 µM sodium selenite (SS), corresponding to 0.45 µM Se, and not supplemented cells (US) were used as positive and negative controls, respectively. To avoid interference due to the vehicle, US and SS-supplemented cells received corresponding amounts of L3 blank samples (i.e., a sample obtained following the in vitro digestion procedure without the addition of any food).

Twenty-four hours after supplementation the medium was removed, centrifuged at 400× *g* for 3 min, and used for lactate dehydrogenase (LDH) release determination. Cells were washed twice with warm DPBS and exposed to Earle’s Balanced Salt Solution (EBSS) (116 mM NaCl, 5.4 mM KCl, 0.8 mM NaH_2_PO_4_, 26 mM NaHCO_3_, 2.38 mM CaCl_2_, 0.39 mM MgSO_4_), containing 1.2 mM H_2_O_2_. Some US cells received the same medium without H_2_O_2_ (US, not stressed cells). After 1 h, EBSS was removed, centrifuged at 400× *g* for 3 min, and used for thiobarbituric acid reactive substances (TBARS) and LDH release determination. In some cells, viability was immediately measured, as described below; other cells were washed twice with cold DBPS, lysed with 1 mL cold Nonidet P-40 (0.25% in DPBS), incubated for 30 min on ice in a shaking platform, and centrifuged at 14,000× *g* for 15 min. The resulting supernatant was immediately stored at −20 °C until further analysis.

### 2.7. Cell Viability

Cell viability was evaluated using the methylthiazolyldiphenyl-tetrazolium bromide (MTT) assay, as reported by Di Nunzio et al. [31]. Cell viability was expressed as relative viability using US, not stressed cells, as the reference.

### 2.8. Lactate Dehydrogenase Activity

LDH activity in the media and EBSS buffer was assessed spectrophotometrically at 340 nm for 1 min measuring the rate of NADH oxidation [32]. Enzyme activity was expressed as mU/mL medium and normalized for mg of protein in the well.

### 2.9. Thiobarbituric Acid Reactive Substances Level

TBARS concentration was assessed in EBSS as previously reported [33]. TBARS level was normalized for protein content and expressed as relative TBARS level using US, not stressed cells, as the reference.

### 2.10. Cytosolic Total Antioxidant Capacity

TAC was evaluated as described above in 100 μL of the cell lysate.

### 2.11. Glutathione Peroxidase Activity

Cytoplasmic GPx activity was assayed using a commercial kit (Cayman Chemical, Ann Arbor, MI, USA). Cells grown in 100 mm dishes were scraped off and lysed in 200 µL of GPx lysis buffer (50 mM Tris-HCl, pH 7.5, 5 mM EDTA, 1 mM DTT, and 1% Triton-X 100). The homogenate was centrifuged at 14,000× *g* for 15 min at 4 °C. GPx activity in the supernatant was measured following the oxidation of NADPH to NADP^+^, due to the reduction of oxidized glutathione. The absorbance change was monitored at 340 nm with a multi-plate reader (Tecan Infinite F200, Männedorf, Switzerland). GPx activity (nmol/min) was calculated using the NADPH extinction coefficient as 0.00622 μM^−1^ cm^−1^, adjusted for the path-length of the solution in the well, and normalized for the protein content of the sample.

### 2.12. Thioredoxine Reductase Activity

Cytoplasmic TrxR activity was assayed using a commercial kit (Cayman Chemical, Ann Arbor, MI, USA). Cells grown in 100 mm dishes were scraped off and lysed in 200 µL of TrxR lysis buffer (50 mM potassium phosphate, pH 7.4, 1 mM EDTA, and 1% Triton-X 100). The homogenate was centrifuged at 14,000× *g* for 15 min at 4 °C. The resulting supernatant was used to determine TrxR activity following the reduction of 5,5′-dithiobis(2-nitrobenzoic acid) to 2-nitro-5-thiobenzoic acid (TNB). The reaction yielded a yellow product that was measured at 405 nm with a multi-plate reader (Tecan Infinite F200, Männedorf, Switzerland). TrxR activity (U/mL) was calculated using the TNB extinction coefficient as 6.35 mM^−1^ cm^−1^, adjusted for the path-length of the solution in the well, and normalized for the protein content of the sample.

### 2.13. Cell Protein Content

Protein content in the cell lysate was determined spectrophotometrically by Comassie assay according to Valli et al. [34], using bovine serum albumin as the standard.

### 2.14. Statistical Analysis

Statistical analysis of the results in the cells was by one-way ANOVA with Dunnet as the post-hoc test (statistically significant results have *p* < 0.05).

## 3. Results

### 3.1. Se Concentration, Bioaccessibility, Speciation, and Total Antioxidant Capacity of Gastro-Intestinal Hydrolysates

Total Se was measured in TQ and L3 fractions. As expected, the type of flour used to make the piadina strongly influenced the total Se concentration, while the type of fermentation had no effect (Table 2). Independently of flour type and fermentation, the total Se content was lower in L3 than in the corresponding TQ fraction.

Se bioaccessibility, calculated as the percent release from the food matrix into the digestive fluids (TQ fraction), was 92.4% for SFCF and 92.7% for SFSF.

The type of fermentation had a clearly detectable effect on the percent content of the different Se forms (Table 3). In fact, in the TQ and L3 fractions of the SFSF samples the content of organic Se (SeMet, with a minor contribution of MeSeCys) was higher than in the SFCF ones.

**Figure 1:**
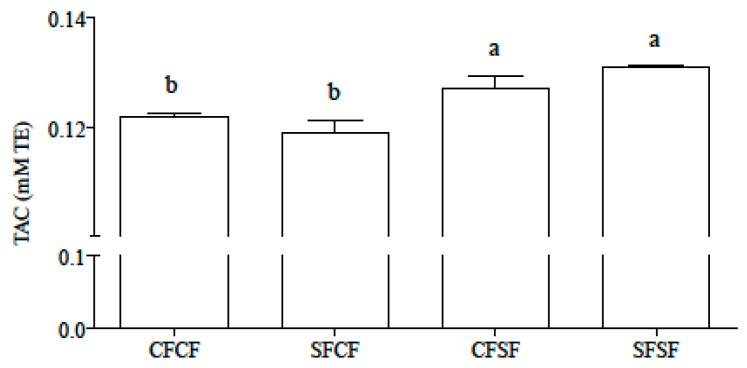
Total antioxidant capacity (TAC) in L3 hydrolysates. Results are expressed as mM Trolox Equivalent (TE) and are means ± SD of triplicate determination. Statistical analysis was by one-way ANOVA (*p* < 0.001), using Tukey’s post-hoc test. Different letters indicate statistically significant differences (at least *p* < 0.05).

TAC of L3 fractions was higher in sourdough than the conventionally fermented piadina, independent of the type of flour used (Figure 1).

### 3.2. Effects in Cultured Cells

**Figure 2:**
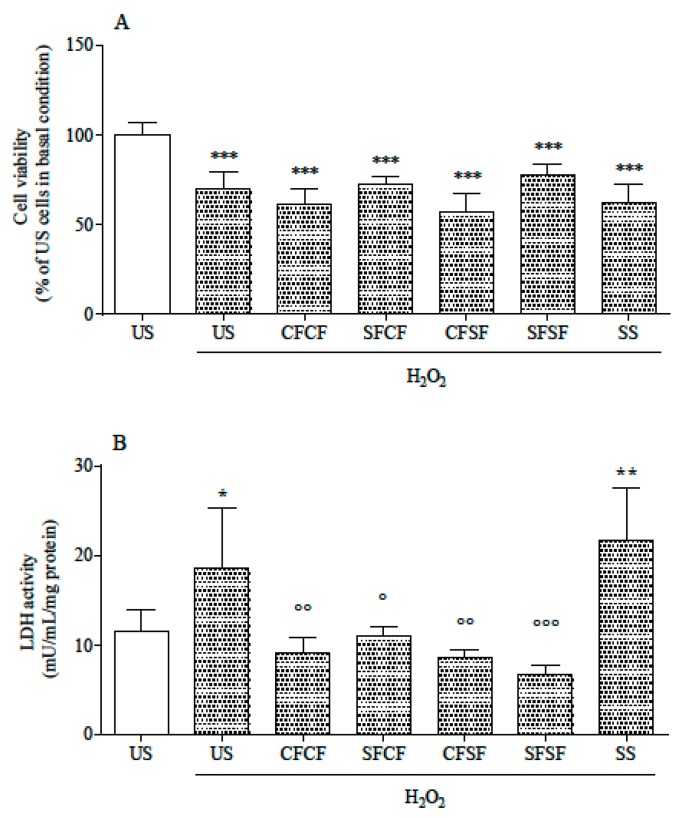
Cell viability (**A**) and lactate dehydrogenase (LDH) release (**B**) in un-supplemented (US) and supplemented cells. Cell viability (panel A) is expressed as % of the viability of US, not stressed cells (assigned as 100%). LDH leakage (panel B) is expressed as mU/mL/mg protein. All data are means ± SD of at least six samples derived from three independent experiments. Statistical analysis was by one-way ANOVA (A and B: *p* < 0.001) using Dunnet’s post-hoc test to compare stressed to US, not stressed cells (* *p* < 0.05; ** *p* < 0.01; *** *p* < 0.001), and supplemented stressed cells to US stressed ones (° *p* < 0.05; °° *p* < 0.01; °°° *p* < 0.001).

In basal conditions, LDH release in the medium was not modified by any supplementation (data not shown), indicating no cytotoxicity. Compared to US, not stressed cells, all stressed cells showed a reduced viability (Figure 2A), whereas LDH release was increased in US and SS-supplemented cells only (Figure 2B). As far as stressed cells were concerned, LDH activity in the medium was significantly lower in supplemented cells than in the US ones, and in the case of SFSF the lowest LDH release was observed.

**Figure 3:**
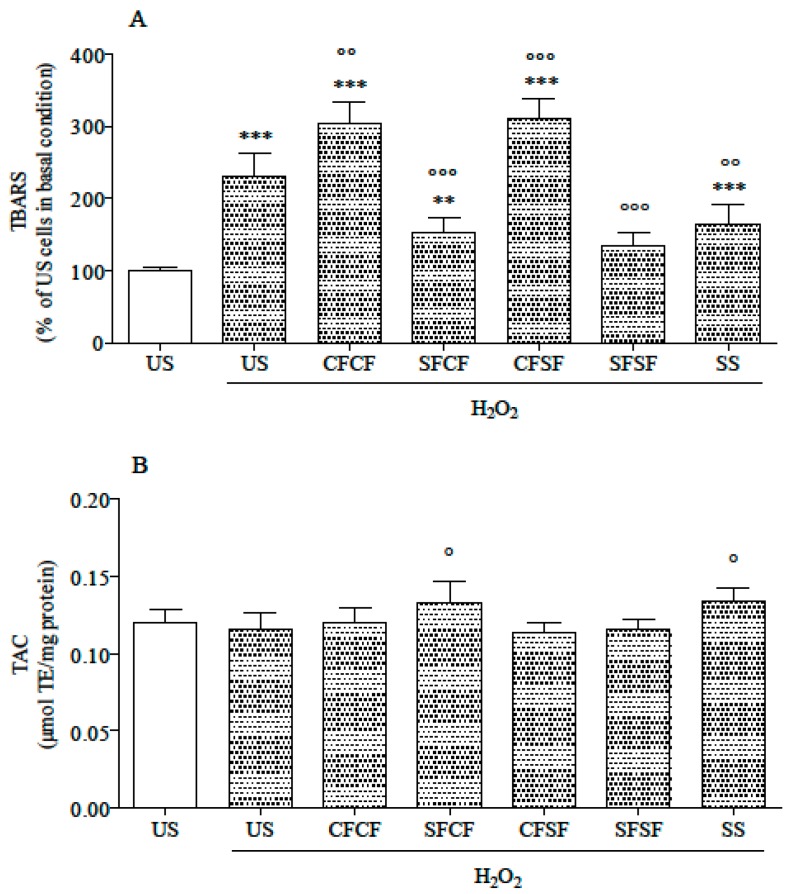
Thiobarbituric acid reactive substances (TBARS) levels (**A**) and cytosolic TAC (**B**) in un-supplemented and supplemented cells. TBARS level (panel A) is expressed as % of the level in US, not stressed cells (assigned as 100%). TAC (panel B) is expressed as µmol TE/mg protein. All data are means ± SD of at least six samples derived from three independent experiments. Statistical analysis was by one-way ANOVA (A: *p* < 0.001; B: *p* < 0.01) using Dunnet’s post-hoc test to compare stressed cells to US, not stressed ones (** *p* < 0.01; *** *p* < 0.001), and supplemented stressed cells to US stressed ones (° *p* < 0.05; °° *p* < 0.01; °°° *p* < 0.001).

Compared to US, not stressed cells, TBARS level significantly increased in all stressed cells except those treated with SFSF (Figure 3A). Among stressed cells, TBARS level was higher in CFCF and CFSF and lower in SFCF, SFSF, and SS than in US cells.

No difference in cytosolic TAC was detected between not stressed and stressed cells, regardless of supplementation (Figure 3B). Among stressed cells, those supplemented with SFCF and SS had the highest TAC level.

**Figure 4:**
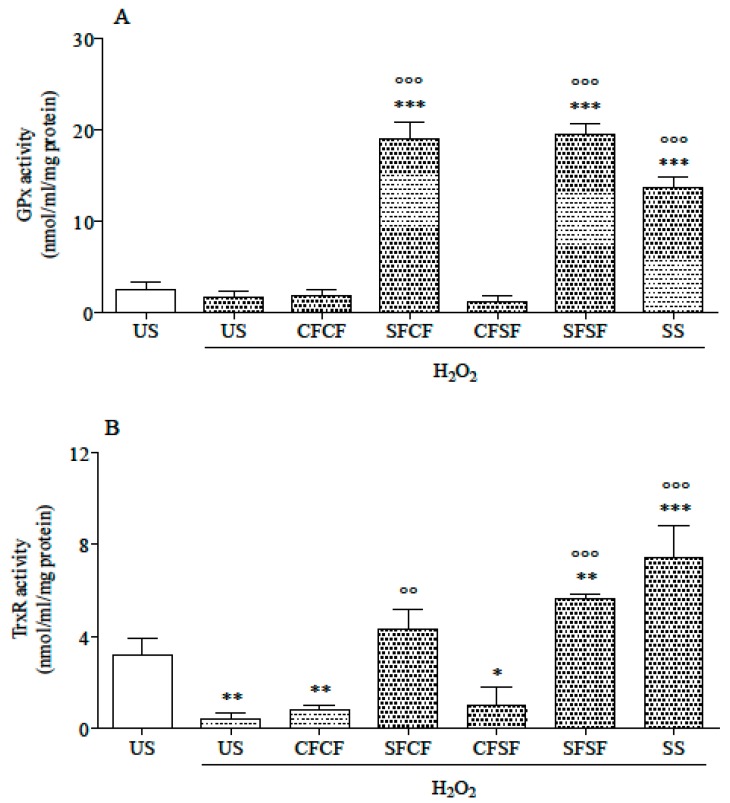
Glutathione peroxidase (**A**) and thioredoxine reductase (**B**) activity. GPx (panel A) and TrxR (panel B) activity is expressed as nmol/mL/mg protein. All data are means ± SD of at least six samples derived from three independent experiments. Statistical analysis was by one-way ANOVA (A: *p* < 0.001; B: *p* < 0.001) using Dunnet’s post-hoc test to compare stressed cells to US, not stressed ones (* *p* < 0.05; ** *p* < 0.01; *** *p* < 0.001), and supplemented stressed cells to US stressed ones (°° *p* < 0.01; °°° *p* < 0.001).

Independently of the source, Se supplementation significantly increased GPx activity, which was higher in SFCF, SFSF, and SS cells than in other cells (Figure 4A). In stressed cells not supplemented with Se, TrxR activity significantly decreased, whereas it was not modified in SFCF and increased in SFSF and SS supplemented cells, compared to US, both stressed and not stressed (Figure 4B).

## 4. Discussion

Bioavailability (i.e., the fractional utilization of a nutrient for normal physiological functions) is a key property to establish the value of a nutrient source, as it goes beyond the mere amount of the nutrient present in the food and focuses on the proportion of the substance that, upon ingestion, is absorbed in a form that is physiologically useful. Bioaccessibility (i.e., the ability of the nutrient to be released from the food matrix and solubilized) is the first component of bioavailability. A good bioaccessibility derives from the results of several factors and critically depends on the disruption of the natural food matrix—or the microstructure created during processing—leading to the release of the nutrient that becomes available for absorption in the digestive tract. In the case of Se, bioavailability critically depends on speciation and it is higher for organic Se compounds [13,14,15,16,17].

In this work we used Se-enriched flour to increase the Se content of piadina and sourdough fermentation, with the aim to promote transformation of inorganic Se into organic forms and increase its bioavailability. Then, the biological effect of the different products was tested by supplementing cultured liver cells with the low molecular weight fraction of the hydrolysates obtained by in vitro piadina digestion.

We found that the use of Se-enriched flour significantly increased the Se content of piadina and this additional Se was largely bioaccesible (≥92%), released from the matrix after in vitro digestion, which was in agreement with previous data on food matrices exhibiting the highest Se bioaccessibility [14]. However, characterization of the Se species in GI hydroxylates showed that only sourdough fermentation, as opposed to conventional fermentation, caused a massive conversion of the inorganic Se present in the flour into organic species. Se speciation in digested piadina prepared with Se-enriched flour was remarkably similar to that of biofortified wheat and derived products [35,36,37], with inorganic Se and MeSeCys as minor species and SeMet, a well-known source of bioavailable organic Se, as major species. SeMet, mainly protein-bound, is also the dominant species (60%–80%) in good quality Se-rich yeasts, followed by SeCys (up to 25%) and a number of minor, low-molecular weight Se metabolites [38].

After in vitro digestion, sourdough-fermented piadina showed a higher TAC compared to piadina prepared using conventional fermentation, regardless of the type of flour used in their preparation. This could be related to the degradation of polyphenols to stronger antioxidant metabolites [39] and/or to the generation of antioxidant bioactive peptides [40], such as GSH [41,42] induced by sourdough fermentation. In line with this hypothesis, in a previous work dealing with fermentation of wheat dough by *L. plantarum* 98A strain, a strong release of polyphenol antioxidant compounds was found [43].

Cell supplementation was performed using the L3 fraction, which contains the Se released from the food matrix and theoretically available for absorption. To evaluate the putative protective antioxidant action of Se supplemented in various forms (SS, SFCF, and SFSF), HepG2 cells were exposed to an oxidative stress. The adverse effects of hydrogen peroxide exposure were evident in US cells, which exhibited a significant decrease in viability and an increase of LDH release and TBARS level. Supplementation, with neither SS nor L3 fractions, fully counteracted the viability decrease, but LDH leakage was reduced in cells supplemented with piadina hydrolysates, compared to US stressed cells, and SFSF was found to be the most effective treatment. In addition, SFSF-supplemented cells showed no TBARS increase in response to oxidative stress. Counteraction of lipid peroxidation was more effective using SFSF, than either SFCF or SS, highlighting that the high concentration of organic Se in SFSF might have played a role.

In biological system, Se antioxidant activity is mainly dependent on the ability to sustain antioxidant activity and reducing enzymes activity, such as GPx and TrxR, and express selenoprotein P [44]. Both in cell culture and in vivo, the availability of Se is a key factor determining GPx and TrxR activity [45,46], which requires an adequate selenium supply [47], and it is lower under selenium-limiting conditions [48].

GPx activity was significantly increased in Se-supplemented cells (SFCF, SFSF, and SS). Comparing the three different Se supplementations, SS appeared to be the least effective one (*p* < 0.001), whereas SFSF was the most active, along with SFCF. Considering that the amount of Se supplemented to cells as SS was higher than those in the L3 fraction, of both SFCF and SFSF, the greater effectiveness of the latter in increasing GPx activity might be due to the different speciation of the supplemented Se, as well as, at least partially, to the so-called “matrix effect” (food hydrolysate vs. pure chemical compound). Bermingham et al. [49], in a comparison of Se-enriched foods and SeMet effectiveness in promoting GPX activity, put forward that Se has a different impact on cell physiology when it is administered as food components or as stand-alone Se-containing molecules. Whatever the cause is, the observed increase in GPX activity after Se supplementation can explain the lower TBARS level in the corresponding stressed cells, as Se-dependent GPx specifically reacts with hydroperoxide [50].

The selenoenzyme TrxR, together with thioredoxin and NADPH, constitute the thioredoxin system, a major cellular redox signaling in almost every living cell [51]. TrxR displays a broad specificity and plays an important antioxidant role, not only by supplying reducing equivalents to the thioredoxin and thioredoxin peroxidase systems, but also by directly reducing H_2_O_2_ and lipid peroxides. In our study, Se supplementation completely prevented the loss of TrxR activity upon oxidative stress. Similarly, previous in vitro studies revealed that Se supplementation reduced oxidative damage and associated genotoxicity by increasing TrxR activity in different cell lines [52,53,54]. On the other hand, Se-dependent antioxidant enzymes have poor specific activity against the radical ions of ABTS, and this could explain the lack of increase in the intracellular TAC for SFSF.

## 5. Conclusions

In several world regions Se dietary intake does not meet the dietary reference values. Se biofortification and the production of Se-enriched food is one of the strategies that can improve human Se nutrition. Our results clearly indicate that this strategy can be further improved by the use of sourdough fermentation of a wheat-based matrix, thus promoting the conversion of inorganic to organic Se. This is of particular importance to the food industry, considering that one of the main drivers of food innovation is the need to find solutions to malnutrition and related health problems.

Herein, reported results highlight that, after simulated human digestion, Se-enriched, sourdough-fermented flatbread counteracts oxidative damage in cultured cells. Although further studies are needed to confirm, in vivo, the antioxidant effect of Se-rich, sourdough-fermented flatbread, our study indicates that increased Se biotransformation towards organic forms is one of the several favorable attributes affected by sourdough fermentation, and which contributes to the enhancement of the nutritional properties of the final product.

## Figures and Tables

**Table 1:** Piadina recipes. CFCF: Control flour and conventional fermentation; SFCF: Se-enriched flour and conventional fermentation; CFSF: Control flour and sourdough fermentation; SFSF: Se-enriched flour and sourdough fermentation.

Ingredients	CFCF	SFCF	CFSF	SFSF
Flour (g)	624.5	624.5	490.8	490.8
Clarified pork fat (g)	62.5	62.5	49	49
Water (mL)	281.7	281.7	4.5	4.5
NaCl (g)	12.9	12.9	10.1	10.1
Starch (g)	18.4	18.4	14.4	14.4
Na_2_SeO_3_ (mg)	-	5.66	-	5.66
Sourdough starter (g)	-	-	431.2	431.2

**Table 2:** Total Selenium (Se) content in gastro-intestinal (GI) hydrolysates.

	CFCF		SFCF		CFSF		SFSF	
	TQ	L3	TQ	L3	TQ	L3	TQ	L3
Total Se (µg/mL)	0.022 ± 0.001	0.006 ± 0.000	0.264 ± 0.004	0.127 ± 0.003	0.012 ± 0.001	0.007 ± 0.000	0.265 ± 0.003	0.109 ± 0.004

Results are expressed as µg/mL and are means ± SD of three technical replicates. TQ: Talis qualis digested sample; L3: fraction containing molecules with molar mass <3 KDa.

**Table 3:** Se speciation: percent distribution of the different Se species in GI hydrolysates.

	Void	MeSeCys (% Total Se)	SeMet (% Total Se)	SeIV (% Total Se)	SeVI (% Total Se)
TQ fraction					
CFCF—TQ	17	5	56	-	21
SFCF—TQ	3	-	14	84	-
CFSF—TQ	13	-	66	-	20
SFSF—TQ	10	2	53	35	-
L3 fraction					
CFCF—L3	10	-	63	-	27
SFCF—L3	2	1	12	85	-
CFSF—L3	8	-	59	-	33
SFSF—L3	9	5	46	40	-

Results are expressed as % of the sum of the detected Se species and are means of three technical replicates. MeSeCys: Se-methylselenocysteine; SeMet: Se-methionine; Selenite: SeIV; Selenate: SeVI.

## Supplementary Materials

The following are available online at http://www.mdpi.com/2072-6643/10/12/1898/s1, Figure S1 and S2: Representative SI chromatograms, Table S1: Performance characteristics of the HPLC-ICP-MS method.
