# Supplementary material for: Sourdough Fermentation Favorably Influences Selenium Biotransformation and the Biological Effects of Flatbread
Source: Nutrients. 2018 Dec 3;10(12):1898. doi: 10.3390/nu10121898 (PMC6316522; doi:10.3390/nu10121898)
Supplement: Supplementary file 1 [file nutrients-10-01898-s001.zip › Di Nunzio et al. Supplementary Material/Figure Caption.docx]

Figure S1. Anion exchange HPLC-ICP-MS chromatogram of selenium species in the SFCF-TQ piadina gastrointestinal hydrolysate.

Figure S2. Anion exchange HPLC-ICP-MS chromatogram of selenium species in the SFSF-TQ piadina gastrointestinal hydrolysate.
