# Supplementary material for: Sourdough Fermentation Favorably Influences Selenium Biotransformation and the Biological Effects of Flatbread
Source: Nutrients. 2018 Dec 3;10(12):1898. doi: 10.3390/nu10121898 (PMC6316522; doi:10.3390/nu10121898)
Supplement: Supplementary file 1 [file nutrients-10-01898-s001.zip › Table S1.docx]

**Table S1**. Performance characteristics of the HPLC-ICP-MS method.

|  | **R***^1^* | **Sensitivity***^2^* | **LOD***^3^* | **LOQ***^4^* | **Accuracy***^5^* |
| --- | --- | --- | --- | --- | --- |
| MeSeCys | 0.993 | 3888 | 0.22 | 0.73 | 93±13 |
| SeMet | 0.995 | 3046 | 0.34 | 1.12 | 96±10 |
| SeIV | 0.998 | 3516 | 0.35 | 1.18 | 112±10 |
| SeVI | 0.995 | 5017 | 0.17 | 0.56 | 87±8 |

*^1^* Correlation coefficient. *^2^* Slope of the calibration curve (a) in the equation *y*= a*x*+b, where *y* is the measured intensity, *x* is the selenium species concentration and b is the intercept set at zero concentration. Expressed as cps. *^3^*Limit of detection expressed as µgSe/L calculated as 3 times the standard deviation of the measurement of 10 method blanks. *^4^*Limit of quantification expressed as µgSe/L calculated as 10 times the standard deviation of the measurement of 10 method blanks. *^5^*Assessed by spiking known amount of Se species at one fortification level, under repeatability conditions. Expressed as percentage.
