# Supplementary figures and images for: Sourdough Fermentation Favorably Influences Selenium Biotransformation and the Biological Effects of Flatbread
Source: Nutrients. 2018 Dec 3;10(12):1898. doi: 10.3390/nu10121898 (PMC6316522; doi:10.3390/nu10121898)

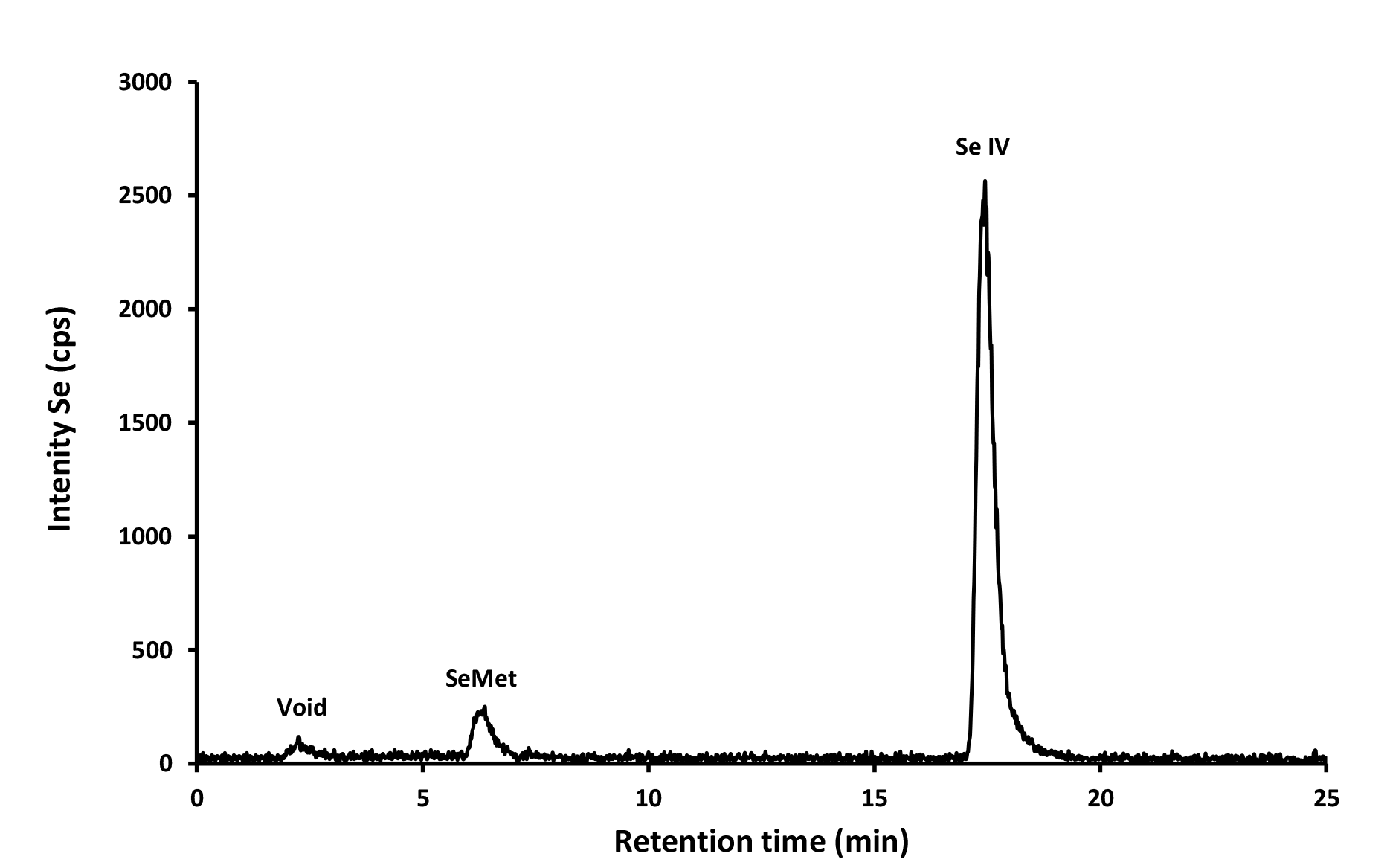

Supplement: Supplementary file 1 [file nutrients-10-01898-s001.zip › Di Nunzio et al. Supplementary Material/Figure S1.tif]

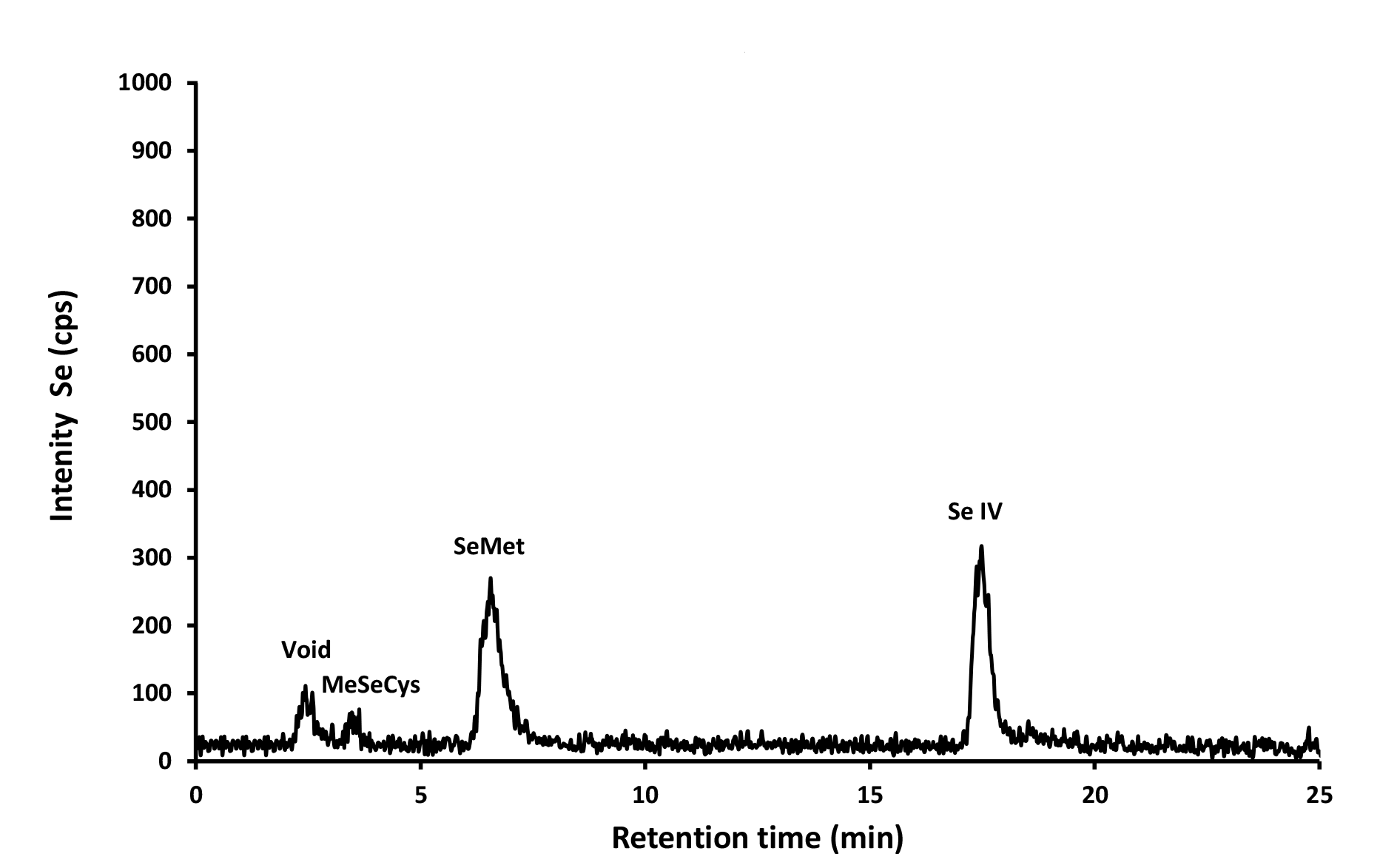

Supplement: Supplementary file 1 [file nutrients-10-01898-s001.zip › Di Nunzio et al. Supplementary Material/Figure S2.tif]
